# The Multifunctional Role of Patatin in Potato Tuber Sink Strength, Starch Biosynthesis, and Stress Adaptation: A Systematic Review

**DOI:** 10.3390/biology15010029

**Published:** 2025-12-24

**Authors:** Yicong Wu, Yunxia Zeng, Wenying Zhang, Yonghong Zhou

**Affiliations:** 1 College of Agronomy and Biotechnology, Southwest University, Chongqing 400700, China; wycwycwycwyc@email.swu.edu.cn (Y.W.); zyx071316@email.swu.edu.cn (Y.Z.); 2 Dryland Farming Institute, Hebei Academy of Agricultural and Forestry Science, Hengshui 053000, China

**Keywords:** abiotic and biotic stress, breeding and biotechnology, patatin, potato tuber sink strength, starch biosynthesis

## Abstract

Potato (*Solanum tuberosum*) is one of the world’s most important food crops, with tuber sink strength and starch deposition determining yield, quality, and processing performance. While starch is the dominant carbohydrate reserve, its accumulation is tightly linked with protein metabolism. Patatin, the major soluble storage protein, constitutes up to 40% of total tuber protein. In addition to serving as a nitrogen and carbon reserve, patatin exhibits lipid acyl hydrolase (phospholipase A_2_-like) activity, suggesting roles in membrane remodeling and stress signaling. This dual identity places patatin at the intersection of storage, metabolic regulation, and defense. A structured review of studies published between 1980 and 2025 was developed using PubMed, Web of Science, Frontiers, and MDPI databases. Prioritized research included molecular, physiological, and multi-omics analyses of patatin expression, regulation, and function under optimal and stress conditions. Evidence indicates that patatin contributes to carbon–nitrogen balance and sink strength by affecting sucrose import, vacuolar osmotic capacity, and starch biosynthesis. Under drought, salinity, and pathogen stress, patatin transcript levels, protein stability, and enzymatic activity shift, leading to reduced starch deposition, altered sugar accumulation, osmoprotection, and reallocation toward defense responses. Despite these insights, major knowledge gaps remain. These include isoform-specific roles, integration into sugar–hormone regulatory networks, and field-scale responses under fluctuating environments. Future progress will require integrated multi-omics, fluxomics, and proximity-labeling approaches, combined with CRISPR-based isoform editing and promoter engineering. Targeting patatin as both a biomarker and an engineering node offers opportunities to develop climate-ready potato cultivars with improved starch yield, tuber quality, and stress resilience.

## Simple Summary

Potatoes are a worldwide staple food, and their tubers store energy and nutrients essential for human consumption. Patatin, a major potato protein, plays multiple roles in tubers, influencing growth, starch storage, and responses to stress. This review brings together studies on patatin to explain how it supports starch production, nutrient storage, and resilience to environmental challenges. By integrating information on metabolism, growth, and stress adaptation, the work provides insights that can guide breeding and crop management strategies. Understanding patatin’s multifunctional roles is valuable for developing potato varieties that are more productive, nutritious, and resistant to stresses such as disease, drought, or extreme temperature.

## 1. Introduction

### 1.1. Tuber as a Carbon Sink

Potato (*Solanum tuberosum* L.) is one of the world’s most widely cultivated and consumed root–tuber crops, with significant nutritional, agronomic, and economic importance. Its high yield potential, adaptability to diverse environments, and role in enhancing food and income security make it a cornerstone in global food systems [1]. Beyond direct consumption, potato supports extensive agro-industrial value chains: potato-derived starches and modified starch products are used in food processing, adhesives, paper production, packaging, and the development of biopolymers [2]. These applications reinforce the tuber’s dual relevance as both a dietary staple and an industrial feedstock. At the physiological level, the tuber serves as a major carbon sink, assimilating, metabolizing, and storing carbohydrates and other assimilates translocated from photosynthetically active leaves. The concept of sink strength is central to this process. Sink strength is generally defined as an organ’s capacity to compete for assimilates from source tissues, and it reflects both sink size (storage capacity) and sink activity (metabolic demand and import kinetics) [3]. Tuber sink strength is dynamic and varies with developmental stage, environmental conditions, and genetic background, and it can be quantitatively expressed as the potential growth rate relative to competing sinks [3].

Several determinants influence tuber sink strength. First, phloem import regulates carbon delivery to the tuber by constraining assimilate flux in accordance with source–sink interactions, transport capacity, and sink demand, rather than establishing an absolute maximum carbon supply. Second, intracellular metabolism establishes the biochemical demand that drives continued assimilate inflow; however, assimilate movement is additionally regulated by hormonal signaling and turgor-driven processes that collectively modulate sink strength. Key enzymes such as sucrose synthase, invertases, and ADP-glucose pyrophosphorylase regulate this process [3]. Third, storage proteins, such as patatin, contribute primarily to nitrogen storage and cell physiology and may influence osmotic homeostasis indirectly through their effects on cellular metabolism and solute balance [4]. Additionally, starch granules stored in amyloplasts constitute the primary long-term carbohydrate reservoir; however, carbon allocation within the tuber also supports the synthesis and accumulation of storage proteins, reflecting a coordinated partitioning of carbon among multiple storage pools. Finally, vacuolar storage capacity and compartmentalization help regulate intracellular sugar concentrations, turgor-driven cell expansion, and metabolite buffering, all of which contribute to overall sink strength [3]. Starch is the predominant carbohydrate reserve in potato tubers and serves dual functions. Nutritionally, it provides a major source of dietary energy, influencing texture and cooking quality. Industrially, native and modified potato starches are valued for their distinctive physicochemical properties, such as high viscosity and paste clarity, with large granule size being characteristic of certain cultivars [2]. Consequently, starch biosynthesis is not only central to tuber development but also to potato’s economic importance, highlighting the importance of understanding the link between sink strength and starch metabolism.

In this context, patatin emerges as a multifunctional protein of particular interest. Traditionally regarded as the principal soluble storage protein in potato tubers, patatin accounts for up to 40% of total soluble protein [5]. Recent studies, however, indicate that patatin is not a passive reserve protein but also exhibits lipid acyl hydrolase and phospholipase A_2_-like activities, linking it to lipid metabolism, membrane remodeling, and stress signaling [4]. This dual identity positions patatin at the interface of lipid metabolism, nitrogen storage, and stress adaptation, with indirect effects on carbon allocation through its influence on cellular metabolism and sink physiology. It is increasingly evident that patatin’s functions extend beyond storage to roles that influence tuber development, sink regulation, and resilience under environmental challenges. Taken together, the global significance of potato, the central role of starch as a dietary and industrial carbohydrate, and the multiscale determinants of sink strength justify a systematic synthesis of patatin’s role in tuber biology. This review critically examines the multifunctional roles of patatin in potato tuber sink strength and stress adaptation. It also explores its contribution to starch biosynthesis. Together, these perspectives provide new insights into how patatin integrates storage, metabolism, and resilience in one of the world’s most important crops.

### 1.2. Patatin: A Brief Historical and Functional Overview

Patatin was first identified in the late 1960s and early 1970s as the predominant soluble protein in potato (*Solanum tuberosum*) tubers, constituting approximately 30–45% of total soluble protein depending on genotype and developmental stage [6]. Early biochemical analyses revealed that patatin accumulates to high levels during tuber development, is deposited in the vacuole, and is readily mobilized during sprouting, leading to its classical designation as a storage protein [7]. The protein is encoded by a multigene family, with estimates of more than 10 homologous genes distributed across the potato genome, allowing both high accumulation and functional diversification [8]. Structurally, patatin belongs to a glycoprotein family with monomers of approximately 40–45 kDa. It is characterized by a conserved patatin domain that defines a wider patatin-like protein (PLP) superfamily, now recognized across plants, fungi, and animals [9]. Within the tuber, patatin is predominantly vacuolar, contributing both to the storage of nitrogen and to cellular osmotic balance. Vacuolar sequestration also protects patatin from premature proteolysis and positions it for rapid mobilization during germination and early seedling growth [7].

### 1.3. Scope, Structure and Methodology of the Review

This systematic review synthesizes current knowledge on patatin from a physiological, biochemical, and agronomic perspective, with particular emphasis on its implications for potato tuber sink strength, starch biosynthesis, and stress adaptation. It aims to synthesize current physiological, biochemical, and agronomic evidence on patatin’s role in potato tuber sink strength, carbon allocation and starch biosynthesis, and stress adaptation. The review is structured around three central questions: (i) Why revisit patatin now? (ii) What are the mechanistic links between carbon allocation and starch metabolism? (iii) How does patatin contribute to stress adaptation in potato tubers? By addressing these questions, this review provides a comprehensive synthesis of patatin’s multifunctionality, positioning it as a central player in the physiological nexus of tuber storage, carbon flux regulation, and stress adaptation. This integrative perspective not only advances fundamental plant biology but also informs applied strategies for crop improvement in an era of increasing demand for sustainable food production and starch-based products.

This systematic review was conducted in accordance with the Preferred Reporting Items for Systematic Reviews and Meta-Analyses (PRISMA 2020) guidelines, which provide a robust framework for transparent, reproducible, and comprehensive evidence synthesis [10]. The approach was carefully designed to capture the breadth of research on patatin, spanning its discovery as a tuber storage protein to its more recently recognized enzymatic functions and roles in tuber sink strength, starch biosynthesis, and stress adaptation. A comprehensive literature search was carried out between May and August 2025 across major scientific databases, including Web of Science, Scopus, PubMed, CAB Abstracts, AGRICOLA, and Google Scholar. The search targeted studies published from 1980, when patatin was first described as a predominant soluble protein in potato tubers, up to 2025. Both peer-reviewed journal articles and high-quality reviews were considered to ensure balanced coverage of foundational and contemporary knowledge. Search strings combined controlled vocabulary and free-text terms such as “patatin,” “patatin-like phospholipase A_2_,” “potato,” “sink strength,” “starch biosynthesis,” “storage protein,” and “stress adaptation,” with Boolean operators used to broaden or refine the queries. Reference lists of key articles were also screened to identify additional relevant publications that were not captured through database searching. To ensure consistency, only peer-reviewed English-language publications were included.

The eligibility of studies was determined through a two-stage screening process. In the first stage, titles and abstracts were reviewed to exclude records unrelated to patatin or potato tuber physiology. In the second stage, full texts of potentially relevant papers were examined to verify alignment with the objectives of the review. Only studies that provided empirical or mechanistic insights into patatin’s storage role, enzymatic activity, or functional relevance to carbon allocation, starch metabolism, or stress responses were included. Articles that lacked primary data, offered speculative commentary without evidence, or focused exclusively on patatin-like proteins from non-plant systems without direct relevance to potato biology were excluded. All eligible studies were then subjected to structured data extraction. A standardized template was developed to record bibliographic details, experimental systems, study design, patatin-related findings, and mechanistic interpretations relevant to sink strength, starch biosynthesis, and stress physiology. Data extraction was undertaken independently by two reviewers, with discrepancies resolved by discussion to reduce subjectivity and potential bias.

Data collection from the included reports was conducted using a structured and standardized data extraction process. Two reviewers independently extracted data from each eligible study using a predefined extraction template that captured bibliographic information, study design, experimental system, patatin-related variables, outcomes, and key methodological details. The independently extracted data were then cross-checked, and any discrepancies were resolved through discussion and consensus. When necessary, original articles were re-examined to verify unclear or missing information; however, no direct contact with study investigators was required. No automation tools or machine-learning–assisted screening or data extraction software were used at any stage of the study selection or data collection process. The overall identification, screening, eligibility assessment, and inclusion of studies are summarized in Supplementary Figure S1. To evaluate the reliability and scientific quality of the included studies, a critical appraisal was performed using an adapted version of the Critical Appraisal Skills Programme (CASP) checklist for experimental plant sciences [11]. This methodological framework provided a structured pathway for integrating four decades of research on patatin. By combining historical perspectives with contemporary molecular insights, the review ensures both breadth and depth in synthesizing the multifunctional roles of patatin within potato tuber physiology.

The risk of bias in the included studies was assessed using an adapted version of the Critical Appraisal Skills Programme (CASP) checklist tailored for experimental plant and physiological research. This tool was selected to allow systematic evaluation of study design clarity, experimental controls, reproducibility, methodological transparency, and the appropriateness of data interpretation. Each included study was independently assessed by two reviewers, and disagreements were resolved through discussion to achieve consensus. No automation tools were used during the risk-of-bias assessment. Given the diversity of experimental designs and outcome measures, studies were grouped for synthesis based on shared conceptual and methodological features rather than identical interventions. Eligibility for each synthesis was determined by tabulating study characteristics, including experimental system (e.g., whole tuber, tissue-specific, molecular assays), type of patatin-related outcome (storage function, enzymatic activity, sink strength indicators, starch metabolism, or stress response), and relevance to the predefined review questions—and comparing these against the planned thematic synthesis framework.

Due to substantial heterogeneity in experimental approaches, outcome definitions, and reporting formats, quantitative effect measures such as risk ratios or pooled mean differences were not consistently applicable, and a formal meta-analysis was not performed. Instead, results were synthesized using a structured narrative approach, with outcomes summarized qualitatively and, where appropriate, semi-quantitatively using reported means, relative changes, or fold differences as presented in the original studies. When summary statistics were incomplete, data were extracted directly from figures or tables when possible, or findings were reported descriptively without numerical aggregation. Results of individual studies were tabulated and visually summarized using comparative tables and conceptual figures to highlight mechanistic patterns and functional relationships. Narrative synthesis was chosen as the most appropriate method given the exploratory and mechanistic nature of the evidence base. Potential sources of heterogeneity, such as developmental stage, tissue specificity, genotype, and stress conditions, were explored through thematic subgrouping rather than formal statistical methods. Sensitivity analyses were conducted qualitatively by examining whether exclusion of studies assessed as having higher risk of bias altered the overall interpretive conclusions, thereby ensuring the robustness of the synthesized findings.

### 1.4. Search Results, Biasness Study Selection, and Characteristics of Included and Excluded Studies

The possible causes of heterogeneity among study results were explored using a structured qualitative approach rather than statistical methods, due to the diversity of experimental designs and outcome measures. Studies were subgrouped thematically according to developmental stage (e.g., tuber initiation versus bulking), tissue or cellular compartment (whole tuber, parenchyma, vacuolar or membrane-associated fractions), experimental context (controlled conditions versus stress treatments), and methodological approach (biochemical assays, molecular analyses, or physiological measurements). Sensitivity analyses were conducted by repeating the narrative synthesis after excluding studies judged to have higher risk of bias or limited methodological transparency, and by comparing conclusions derived from older foundational studies versus more recent molecular investigations; these analyses did not materially alter the overall interpretive trends. The risk of bias due to missing results or selective reporting was assessed qualitatively by examining consistency of outcome reporting across studies and publication periods, and no strong evidence of systematic non-reporting of negative or null findings was identified, although moderate reporting bias could not be fully excluded. Finally, certainty in the body of evidence for each major outcome was evaluated using a structured, narrative adaptation of evidence confidence frameworks, considering study quality, consistency of findings, biological plausibility, and coherence across independent lines of evidence; overall confidence was rated as high for patatin’s role as a tuber storage protein and moderate for its integrative roles in sink strength regulation, starch biosynthesis, and stress adaptation.

The literature search and study selection process is summarized in Supplementary Figure S1 (PRISMA flow diagram). A total of 600 records were identified through database searches and reference list screening. After removal of 220 duplicate records, 380 unique records were screened based on titles and abstracts. Of these, 259 records were excluded for lack of relevance to patatin or potato tuber biology, leaving 121 full-text articles assessed for eligibility. Following full-text evaluation, 27 articles were excluded for specific reasons, including non-experimental or review-only articles (*n* = 11), studies lacking valid patatin-related data or interpretable outcomes (*n* = 10), and duplicate reports of the same datasets (*n* = 6). Ultimately, 94 studies met the inclusion criteria and were included in the qualitative synthesis. The included studies spanned a wide temporal range and encompassed diverse experimental systems, including whole-tuber analyses, tissue- and cell-specific assays, biochemical enzyme characterizations, molecular expression studies, and stress-response experiments across multiple potato cultivars and developmental stages. A full citation list and detailed characteristics of each included study—including experimental design, patatin-related outcomes, and contextual variables—are provided in the Supplementary Table S1.

### 1.5. Risk of Bias, Synthesis Outcomes, and Certainty of Evidence

Risk-of-bias assessments for individual studies are presented in the Supplementary Materials and summarized across thematic syntheses. Overall, most studies exhibited low to moderate risk of bias, with limitations primarily related to incomplete reporting of experimental replication, environmental controls, or statistical methods rather than systematic methodological flaws. Given the substantial heterogeneity in outcome measures and reporting formats, quantitative effect estimates with confidence intervals were inconsistently available across studies, precluding formal meta-analysis. Instead, outcome data were presented using structured tables summarizing reported means, relative changes, fold differences, or qualitative directional effects, accompanied by narrative interpretation. For each thematic synthesis—covering patatin’s role in storage, enzymatic activity, sink strength regulation, starch biosynthesis, and stress adaptation—the contributing studies generally showed consistent directional trends despite methodological diversity. Heterogeneity was explored qualitatively by comparing outcomes across developmental stages, tissue types, genotypes, and stress conditions, revealing context-dependent effects of patatin function. Sensitivity analyses, conducted by excluding studies assessed as having higher risk of bias or limited methodological detail, did not materially alter the overall conclusions. Potential reporting bias was assessed qualitatively and judged to be low to moderate, as the literature included both positive and neutral findings across decades of research. Taken together, the certainty of evidence was rated as moderate to high for patatin’s role as a major storage protein and enzymatic contributor to tuber physiology, and moderate for its proposed integrative roles in sink strength regulation, starch metabolism, and stress adaptation, reflecting strong biological plausibility supported by convergent but methodologically diverse evidence.

## 2. Patatin Gene Family, Protein Architecture, and Cellular Trafficking

### 2.1. Gene Family Organization

The patatin gene family is one of the most abundant and functionally diverse protein groups in potato (*Solanum tuberosum* L.), consisting of approximately 40–45 members distributed across multiple chromosomes [12,13]. Despite their shared sequence homology, patatin genes are categorized into two distinct subfamilies, Class I and Class II based on their regulatory features, expression patterns, and potential functional specializations. Class I patatin genes represent the major storage protein fraction, contributing up to 80% of the total patatin transcript levels (mRNA) expressed during tuber development [13]. Their transcription is strongly induced during tuber expansion and starch accumulation, reflecting their central role in determining tuber sink strength and carbon allocation. Class I genes are highly sucrose-inducible, ensuring that their expression responds dynamically to carbohydrate availability in the phloem during tuber filling [14]. By contrast, Class II genes are expressed at lower levels and exhibit less tissue specificity, being detected not only in tubers but also in vegetative organs such as leaves and stems [15]. While Class I isoforms function predominantly as vacuolar storage proteins, Class II isoforms retain greater enzymatic potential, particularly lipid acyl hydrolase-like activity, which has been implicated in lipid metabolism and defense responses under stress conditions [16].

At the genomic level, patatin genes are dispersed across several chromosomal regions, with clusters identified in specific loci that highlight their evolutionary expansion through gene duplication events [12]. Considerable copy-number variation (CNV), which refers to gene copy number and alleles in polyploid cultivars, exists among potato cultivars, reflecting the polyploid nature of the crop and its extensive allelic diversity. Proteomic analyses have revealed between 17 and 23 distinct patatin isoforms per cultivar, and this diversity correlates with key agronomic and nutritional traits, including starch content, dry matter percentage, and amino acid balance [17]. The structural organization of these genes typically involves multiple introns and exons in most studied cultivars, with Class I patatin genes commonly containing nine exons and eight introns, while Class II genes often display fewer introns. This structural diversity likely facilitates alternative splicing, which expands the isoform repertoire and supports both storage and enzymatic functions [18]. Patatin gene expression is tightly regulated by cis-regulatory elements in their promoters, which integrate metabolic, developmental, and hormonal cues. The best characterized is the B33 promoter of Class I patatin, which has been widely employed in transgenic research to drive tuber-specific expression of recombinant proteins and engineered metabolic pathways [13]. Promoter analyses have identified sucrose-responsive elements (SUREs), which mediate transcriptional activation under elevated sucrose levels, linking patatin expression to carbohydrate supply [19]. Additionally, abscisic acid (ABA)-responsive elements (ABREs) enhance patatin transcription during tuberization, highlighting the role of hormonal regulation in sink differentiation [20]. Other tuber-specific elements confer strong expression confined to developing tubers, ensuring the preferential deposition of patatin in storage vacuoles. The combination of sucrose sensitivity, hormonal responsiveness, and tissue specificity makes patatin promoters essential molecular switches in the regulation of tuber sink metabolism and invaluable tools for biotechnological applications [14].

### 2.2. Protein Biochemistry and Post-Translational Features

The biochemical identity of patatin is shaped by a conserved patatin domain that confers both storage-protein properties and enzymatic capacity, producing a molecule that bridges reserve accumulation and metabolic activity in the tuber. Contemporary comparative and structural analyses describe the patatin fold as centered on a nucleophilic serine and an acidic partner (a Ser–Asp catalytic dyad) rather than the classical Ser–His–Asp triad of many canonical lipases; this arrangement underlies the protein’s intrinsic lipid acyl hydrolase (LAH) or phospholipase A_2_ (PLA)-like activity and explains the mechanistic flexibility seen across isoforms [21,22]. Patatin-type enzymes do not require Ca^2+^ for catalysis a key functional distinction from many secreted PLA_2_ enzymes, and biochemical surveys of patatin family members consistently report Ca^2+^-independent activity, which has important consequences for how patatin operates in the relatively low and stable Ca^2+^ concentration in the vacuole [21,23]. Substrate scope and catalytic behaviour are isoform- and context-dependent. Patatin exhibits broad substrate tolerance in vitro, hydrolyzing glycerophospholipids (including phosphatidylcholine and phosphatidylethanolamine), lysophospholipids, and neutral acylglycerols to release free fatty acids and lysolipids; however, individual isoforms often display measurable preferences for chain length and positional specificity that depend on acyl-chain presentation, micellar versus membrane substrates, and assay conditions [16,23]. Recent protein-engineering studies demonstrate this plasticity: rational mutagenesis produced patatin variants with markedly enhanced activity toward long-chain substrates and altered pH and thermal optima, indicating that modest sequence changes around the active site or substrate-binding cleft can retune substrate preference and stability. Reported pH optima for native and engineered patatins typically fall in the neutral to mildly acidic range (approximately pH 6–8) in vitro studies, consistent with activity in the vacuolar microenvironment and with functional roles spanning storage and stress-response contexts [16,23].

The life history of a patatin polypeptide is shaped by multiple post-translational events that govern folding, trafficking, stability, and eventual mobilization. Patatin precursors are synthesized with an N-terminal signal peptide that directs co-translational insertion into the endoplasmic reticulum (ER), where signal peptide cleavage and initial N-linked glycosylation take place; these early modifications are crucial for correct folding and for engagement with the secretory pathway [21,24]. Subsequent glycan maturation in the Golgi and interactions with vacuolar sorting machinery facilitate delivery to protein storage vacuoles (PSVs). Glycosylation itself is functionally important: conserved N-glycosylation sites influence solubility, resistance to proteolysis, and oligomerization propensity, and differential glycosylation among isoforms contributes to the biochemical heterogeneity observed in proteomic surveys [17,21]. Vacuolar residence is associated with further structural transitions. Within PSVs patatin is commonly detected both as soluble monomers (~40–45 kDa) and as higher-order assemblies or storage bodies. Proteomic and imaging studies document oligomeric species and dense protein aggregates that likely arise from non-covalent associations stabilized by glycan interactions, ionic conditions, and protein–protein contacts; such assemblies serve to concentrate reserve protein while modulating access by vacuolar proteases [17,22]. The degree of oligomerization influences functional outcomes: highly aggregated or glycan-stabilized pools tend to be more protease-resistant and thus longer-lived during dormancy, whereas more labile isoforms often those that are post-translationally modified by phosphorylation are primed for rapid mobilization [18].

Mobilization of patatin during sprouting or under stress is a multistep, regulated process. Phosphorylation has emerged as a key post-translational signal that marks specific patatin isoforms for degradation; phosphoproteomic studies show increased phosphorylation of certain isoforms as tubers exit dormancy, with phosphorylated forms preferentially targeted by vacuolar cysteine proteases and other hydrolases [18]. Concomitantly, changes in vacuolar pH, redox state, and protease activity (itself regulated by developmental and stress cues) accelerate proteolysis, releasing amino acids and fatty acid derivatives that feed the energetic and biosynthetic demands of the sprout or that act as signaling molecules during abiotic or biotic stress responses [21,22]. Protein-engineering work showing that specific substitutions enhance thermal stability and reduce susceptibility to proteolysis further supports the link between primary sequence, post-translational state, and functional longevity in storage vacuoles [23].

### 2.3. Cellular Localization and Turnover

**Figure 1:**
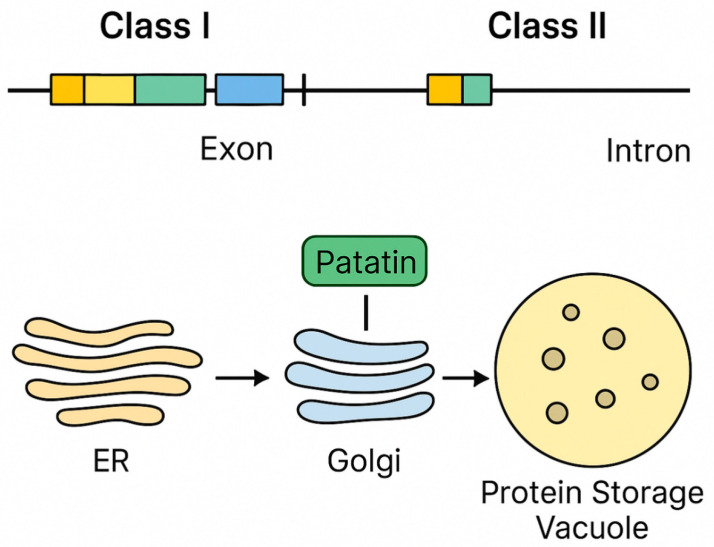
(Schematic): Patatin gene family and protein domain map; trafficking to protein storage vacuoles (PSVs).

Patatin, like other major vacuolar storage proteins in higher plants, follows a highly regulated intracellular trafficking pathway that ensures its correct localization in tuber parenchyma cells. The biosynthesis of patatin begins in the endoplasmic reticulum (ER), where the protein is synthesized as a pre-protein containing a signal peptide that directs it into the ER lumen as represented in Figure 1 and Table 1. Following co-translational translocation, patatin undergoes initial folding and glycosylation before being transported through the Golgi apparatus, where it may receive further glycan modifications (N- and O-glycosylation depending on isoform). From the Golgi, patatin is sorted and targeted to the protein storage vacuole (PSV), a specialized vacuolar compartment that serves as the primary site for reserve protein deposition in developing potato tubers [25,26]. The accumulation of patatin within PSVs is tightly linked to tuber development and the establishment of sink strength. During this stage, patatin aggregates into protein bodies, where it is in close proximity with other vacuolar proteins and starch granules, contributing to the structural stability of the storage organelle [27]. The vacuolar deposition of patatin not only provides a nitrogen and carbon reserve but also may contribute indirectly to osmotic homeostasis. Studies using immunolocalization and fluorescent tagging have confirmed that patatin follows the canonical ER–Golgi–PSV trafficking route and accumulates in dense protein aggregates that remain relatively inert until remobilization is triggered [28].

Turnover of patatin occurs predominantly during tuber sprouting and germination, when the dormant storage organ transitions into a source tissue. At this stage, proteolytic enzymes such as cysteine proteases are upregulated, leading to the hydrolysis of patatin and the release of amino acids and lipid-derived metabolites required to support the growth of emerging shoots and roots [29]. Proteolysis is selective and developmentally regulated, ensuring that degradation coincides with metabolic shifts associated with dormancy release. In addition to developmental turnover, patatin is also subject to stress-induced mobilization. Under abiotic stresses such as drought or cold, or during pathogen attack, patatin can be degraded more rapidly, providing both metabolic intermediates and, in some cases, production of free fatty acids that contribute to stress adaptation and defense responses [23,30].

**Table 1:** Representative patatin genes/isoforms, promoter features, and protein properties. N/A indicates not applicable.

Patatin Gene/Isoform	Class	Promoter Features	Protein Size/Domain Features	Localization	Notes/Functions
StPat I (e.g., PGSC0003DMG 400012345)	Class I	Strong tuber-specific promoter; sucrose-responsive elements (SURE); ABA-responsive motifs (ABRE) [14]	~43 kDa; conserved patatin domain with Gly-X-Ser-X-Gly catalytic motif [23]	Vacuole (PSVs) [18]	Highly expressed in tubers; widely used in transgenic studies for tuber-specific expression [14]
StPat II (e.g., PGSC0003DM G400045678)	Class II	Weaker promoter activity; less tuber-specific; inducible by stress (ABA, drought) [31]	~40–42 kDa; PLA_2_-like domain [32]	Vacuole; partial cytoplasmic aggregates [33]	Stress-associated isoform; contributes to lipid acyl hydrolase activity under abiotic stress [32]
StPat Class I promoter (synthetic constructs)	Class I	Contains SURE, ABRE, and TATA-like elements; high specificity to tuber parenchyma [14]	Used in gene constructs rather than direct protein product	Drives tuber-specific recombinant expression [18]	Widely used in potato biotechnology and heterologous protein expression [14]
StPat II isoform variants	Class II	Promoter enriched in ABA/ethylene-responsive motifs; low sucrose response [31]	~41 kDa; isoforms with post-translational glycosylation variability [32]	Vacuole and transient ER retention [33]	Suggested role in stress adaptation and remobilization during sprouting [32]
Chimeric Patatin Promoters (e.g., B33)	Derived from Class I	Engineered for high tuber expression; retains SURE and ABRE motifs [14,18]	Not a protein product (promoter only)	N/A	Extensively used in metabolic engineering of potato tubers [18]

## 3. Spatiotemporal Regulation of Patatin During Tuber Development

### 3.1. Developmental Timeline

Patatin expression in potato tubers follows a distinct spatiotemporal pattern that closely parallels starch deposition during development. During stolon swelling and tuber initiation, patatin transcripts are detectable but remain at relatively low levels compared to later stages [15]. As tubers transition into the bulking phase, expression of patatin genes increases markedly, reflecting the high demand for both storage proteins and structural sink capacity [8]. This bulking phase coincides with maximal sucrose import and the steepest rise in starch accumulation, suggesting a coordinated regulation between protein and carbohydrate reserves [34]. Patatin proteins accumulate predominantly in the vacuole, forming protein storage vacuoles that expand in parallel with amyloplasts, thereby supporting tuber sink strength [35]. During maturation, patatin content reaches its peak, comprising up to 40% of total soluble protein, before declining slightly as tubers enter dormancy or sprouting phases [36]. Comparative analyses of starch curves and patatin accumulation reinforce the view that patatin is not a passive storage component but actively integrated into the developmental program of the potato tuber.

### 3.2. Regulatory Inputs

The regulation of patatin expression is highly responsive to carbohydrate signaling, hormonal inputs, and developmental cues. Sucrose is the dominant signal for patatin induction, acting through both metabolic and transcriptional pathways [19]. Evidence suggests that sucrose-mediated patatin activation is coupled to trehalose-6-phosphate (T6P) signaling and SnRK1 activity, which function as integrators of sucrose status and energy balance in tuber tissues [37,38]. Hormonal crosstalk further modulates this regulation: abscisic acid (ABA) enhances patatin expression under water-limiting conditions, while gibberellins (GA) can repress tuber-specific promoters, linking patatin regulation to stress responses and developmental transitions [39]. Cytokinins and auxin contribute to the timing of tuber initiation and bulking, with downstream effects on patatin accumulation through their modulation of sink development and vascular unloading [40]. Moreover, tuberization is regulated by long-distance signals such as StSP6A, a florigen-like protein, which has been shown to interact with carbohydrate metabolism pathways [41]. While transcriptional regulation is well established, epigenetic mechanisms remain underexplored. Some evidence indicates that histone modifications and chromatin remodeling may influence the responsiveness of patatin promoters to sucrose and ABA, but systematic studies are lacking [42]. The epigenetic dimension thus represents a promising frontier in understanding how patatin integrates environmental signals with tuber developmental programming.

### 3.3. Co-Expression and Network Context

**Figure 2:**
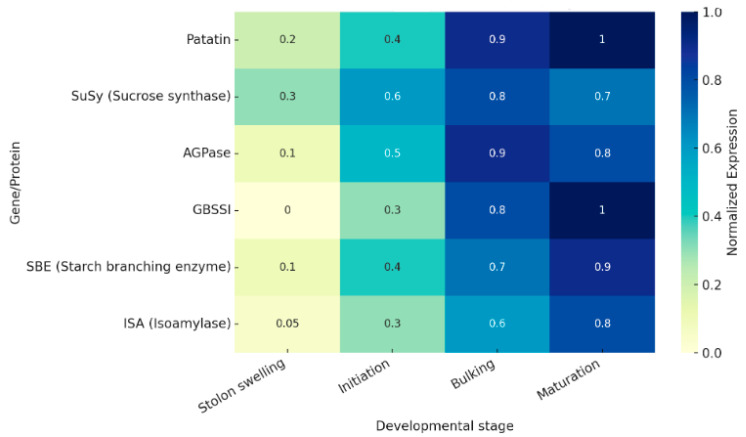
Heatmap or line plots showing patatin vs. starch enzyme expression across stages from published datasets.

Network analyses have revealed that patatin genes co-express with starch biosynthetic modules (Figure 2), supporting the hypothesis that protein and carbohydrate storage are co-regulated processes. Patatin expression clusters with key enzymes of starch biosynthesis, including sucrose synthase (SuSy), ADP-glucose pyrophosphorylase (AGPase), granule-bound starch synthase I (GBSSI), and the starch branching (SBE) and debranching enzyme (ISA) families [43,44]. This co-expression suggests shared transcriptional regulators and common promoter motifs responsive to sucrose and hormonal cues [20]. Patatin-specific promoters have long been exploited in transgenic research for tuber-targeted expression, further demonstrating their developmental synchronization with starch pathways [45]. In addition, systems-level studies have identified sink identity markers genes that distinguish storage sinks such as tubers from vegetative tissues showing that patatin is embedded within a broader regulatory network defining tuber sink strength [46]. Co-expression data also imply that patatin may indirectly influence starch deposition by shaping vacuolar storage dynamics and contributing to nitrogen–carbon balance, thereby affecting carbohydrate partitioning. Integrative models now position patatin as a nodal point in the sink regulatory network, bridging protein reserves with starch biosynthesis and stress adaptation.

## 4. Patatin in Carbon Flux Allocation and Sink Strength

### 4.1. Conceptual Links Between Storage Protein and Carbon Economy

In potato tubers, patatin represents not only a major storage protein but also a critical determinant of carbon–nitrogen (C:N) balance within the sink. As a vacuolar protein, patatin provides a nitrogen reservoir that complements the large carbon stores in starch granules, creating a balanced supply of amino acids and carbohydrates for sprouting and early growth [47]. This dual function highlights vacuolar storage capacity as an often-overlooked component of sink strength, working in parallel with starch granule formation [48]. Recent findings suggest that storage proteins such as patatin can influence carbohydrate partitioning by modulating osmotic potential and cellular expansion in developing tubers [49]. Thus, patatin integrates into the carbon economy by acting both as a nitrogen buffer and a vacuolar structural factor supporting sustained sink activity.

### 4.2. Mechanistic Hypotheses

Two working hypotheses have been proposed to explain patatin’s mechanistic role in carbon allocation. H1 posits that patatin accumulation itself enhances sink strength by maintaining vacuolar osmotic capacity, thereby sustaining sucrose import and indirectly stimulating starch biosynthesis [49]. In this model, patatin acts as a passive enhancer of carbon flux. Conversely, H2 suggests an active sugar–protein feedback, where sucrose signaling pathways mediated by trehalose-6-phosphate (T6P) and SnRK1 regulate patatin expression, while patatin’s nitrogen storage and remobilization reciprocally influence carbohydrate metabolism [50]. This hypothesis aligns with evidence that redox-mediated activation of ADP-glucose pyrophosphorylase (AGPase) operates in tandem with sugar and nitrogen signaling [51]. Together, these models frame patatin as both a consequence and a driver of carbon flux reprogramming in tubers.

### 4.3. Evidence Landscape

**Figure 3:**
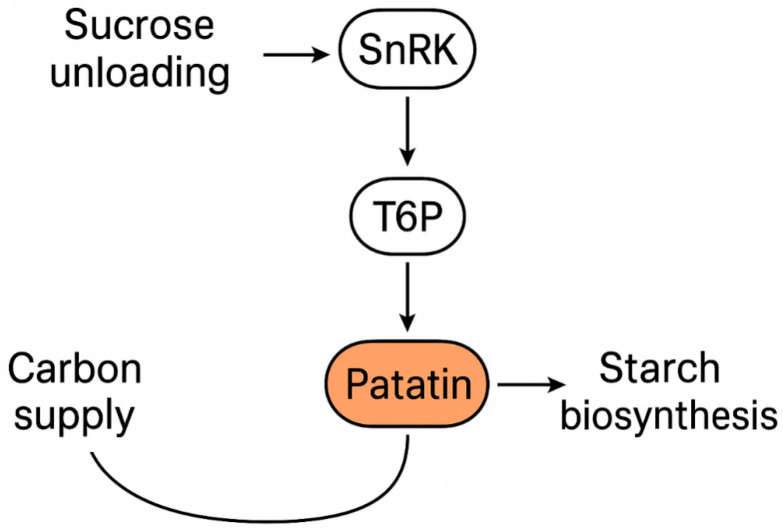
Systems diagram linking sucrose unloading, sugar signals (T6P/SnRK1), patatin accumulation, and starch biosynthetic control points.

Recent datasets provide emerging evidence to support these hypotheses (Figure 3). As presented in Table 2, greenhouse and field studies reveal strong correlations between patatin accumulation and starch deposition curves across cultivars, suggesting coordinated regulation of storage protein and carbohydrate metabolism [52]. Perturbation experiments using overexpression and CRISPR/Cas9 knockouts of patatin genes demonstrate shifts in tuber dry matter and sucrose-to-starch conversion efficiency [53]. Isotopic fluxomics using ^13^C and ^15^N labeling has further shown that patatin influences nitrogen remobilization during sprouting, linking protein turnover with carbon export from tubers [48]. Proteolysis of patatin during dormancy release highlights its dual role: first as a sink-strength stabilizer during development, and later as a source of nitrogen and carbon skeletons fueling sprout growth [54]. Collectively, these findings position patatin as a dynamic regulator of sink capacity, influencing both the magnitude and timing of carbohydrate flux in potato tubers. Table 2 summarizes diverse approaches ranging from systems biology, transcriptomics, and proteomics to gene editing, highlighting how patatin abundance or manipulation correlates with starch yield and composition in potato tubers.

## 5. Interfaces with Starch Biosynthesis

### 5.1. Starch Pathway Refresher

The core routes that supply precursors for starch synthesis in storage organs remain conceptually the same but recent work clarifies their tissue-specific contributions. Sucrose delivered to the tuber apoplast can be metabolized either by sucrose synthase (SuSy) which produces UDP-glucose and fructose and is often associated with anabolic sink metabolism or by invertases, which irreversibly cleave sucrose into glucose and fructose and are implicated in hexose signaling and osmotic regulation; the relative importance of these routes varies by tissue, developmental stage, and species [59,60]. Downstream, ADP-glucose pyrophosphorylase (AGPase) remains the central gateway enzyme producing ADP-glucose for starch polymerization and is a major regulatory node subject to allosteric and post-translational control [61,62]. Chain elongation is catalyzed by granule-bound starch synthase I (GBSSI) for amylose and by soluble starch synthases for amylopectin, while branching and debranching enzymes (SBE, ISA families) shape granule architecture and the amylose:amylopectin ratio [63]. Recent reviews emphasize that regulatory control is multi-layered transcriptional, post-translational (including redox and phosphorylation), and metabolite-mediated and that enzyme isoforms show tissue- and developmental specificity that is critical for final starch structure in tubers [1,63].

### 5.2. How Patatin Modulate the Pathway

There are two non-exclusive mechanistic routes by which patatin may influence starch metabolism. First, spatial coupling: protein storage vacuoles (PSVs) and amyloplasts are often closely juxtaposed in storage parenchyma, and emerging ultrastructural and cell-biological studies show dynamic plastid–vacuole contact zones and membrane contact sites that can permit metabolite and lipid exchange [64,65]. Such contacts create plausible microdomains where patatin (localized to PSVs) could affect local substrate pools or signaling lipids that influence amyloplast enzyme activity or substrate availability. Second, lipid-mediated signaling: patatin has PLA-like activity (patatin-family PLAs/pPLAs) and can release free fatty acids that are substrates for lipoxygenases, producing oxylipins (e.g., jasmonates and related oxidized lipids) known to modulate plastid metabolism and stress responses [4,66]. Lipid signals derived from PLA activity may therefore act as short-range signals that alter plastid redox state, kinase activities (including SnRK1 interactions reported to affect AGPase) or membrane properties that influence starch enzyme localization/function. Finally, on a resource-economics level, proteostasis and resource allocation during bulking require ATP and reducing power for both starch biosynthesis in amyloplasts and protein folding/processing in the secretory–vacuolar system; competition or coordinated regulation of these energy sinks (and their chaperone/protease systems) could shift carbon partitioning between starch and storage protein [67,68]. Thus, patatin could modulate starch biosynthesis both by generating lipid signals that influence plastidial enzyme regulation and by shaping cellular energy/nitrogen budgets that determine flux into ADP-glucose and polymerization.

### 5.3. Data Gaps and Testable Predictions

Despite plausible mechanistic links, direct causal evidence linking patatin isoforms to specific starch metabolic outcomes in potato tubers remains sparse. Key gaps include isoform-specific functions (many patatin genes/isoforms exist and may differ in PLA activity, vacuolar targeting, or stress inducibility), lacking high-resolution interactomes that place patatin physically or functionally near amyloplast proteins, and limited post-translational mapping (e.g., phosphorylation or redox modifications) of patatin and starch enzymes under comparable developmental stages or stresses [65,69]. Testable predictions and experimental strategies to close these gaps need to include: (1) isoform-resolved genetic perturbation (CRISPR/Cas targeting single vs. multiple patatin genes) combined with high-resolution starch phenotyping (granule size, amylose:amylopectin, crystallinity); (2) proximity labeling (TurboID/APEX) from PSV-localized bait proteins to capture proteins and lipids at PSV-amyloplast contact zones; (3) integrated lipidomics + phosphoproteomics following inducible alteration of patatin activity to detect early signaling lipids and downstream kinase changes that affect AGPase/GBSSI activity; and (4) dual fluxomics (^13^C for carbon, ^15^N for nitrogen) during bulking in patatin perturbants to quantify shifts in partitioning [2,70]. Methodological advances proximity labeling in plant storage tissues, improved plastid–vacuole contact imaging, and sensitive oxylipin lipidomics now make these experiments tractable and would directly test whether patatin functions as a signaling nexus between vacuolar protein storage and amyloplast starch synthesis (Table 3).

## 6. Patatin Under Abiotic and Biotic Stresses

### 6.1. Drought

Drought markedly reprograms tuber metabolism, and patatin expression and abundance respond dynamically to water deficit in ways that can affect carbohydrate partitioning. Transcriptome and proteome analyses in potato cultivars under controlled drought show widespread changes in genes and proteins involved in starch and sucrose metabolism together with altered expression of vacuolar/storage proteins [52,74]. Patatin-like proteins (pPLAs) have PLA-like activity and are well placed to affect membrane lipid composition under drought: PLA activity releases free fatty acids that are precursors for oxylipin signaling (e.g., jasmonates and other oxidized lipids) which can modify plastid function and stress responses [4]. Drought also elevates ABA and ABA-dependent signaling cascades that both conserve water and reprogram sink metabolism; ABA signaling has been linked to changes in storage protein gene expression in storage organs [52]. Functionally, drought often causes a shift from starch accumulation toward soluble sugars (osmoprotectants) in tubers a response that could be reinforced if drought downregulates patatin accumulation or activates patatin proteolysis, thereby altering the local C:N balance and the vacuolar osmotic environment [52,74]. In short, drought can modulate patatin at transcript and protein levels, patatin/pPLA activity can remodel membranes and generate lipid signals, and these changes feedback on the starch versus sugar outcome that determines osmoprotection and processing quality [4,52,74].

### 6.2. Salinity

Salinity imposes both osmotic and ionic stresses that perturb vacuolar homeostasis and proteostasis; proteomic surveys under salt stress identify remodeling of storage proteins and protein-folding/degradation machineries [75]. High salt can destabilize vacuolar protein storage compartments or change their protease activity, hastening degradation of storage proteins and releasing amino acids a process that can lower tuber dry matter and alter starch content and quality [75]. Because patatin is a major vacuolar constituent, salinity-driven shifts in vacuolar pH, ionic composition, or protease activation could accelerate patatin turnover, thereby altering the tuber’s nitrogen reserve and indirectly influencing carbohydrate partitioning. Additionally, salt stress interacts with ROS and lipid peroxidation pathways; if patatin/pPLA activity changes under salinity, altered lipid signaling may compound effects on plastid metabolism and starch biosynthesis [4,75]. Empirically, salt-affected tubers often display reduced dry matter and starch concentration, consistent with a model where vacuolar protein instability and resource reallocation contribute to quality losses under salinity [75].

### 6.3. Pathogen Attack (Fungi, Bacteria, Viruses) and Wounding

Patatin and patatin-derived peptides have been reported to exhibit antimicrobial properties and can be part of wound- and pathogen-induced responses [76]. Wounding and pathogen challenge rapidly alter local proteomes and the apoplastic/extracellular milieu; patatin and patatin-derived peptides released during proteolysis may act directly on microbial invaders or function indirectly via generation of lipid mediators when PLA activity liberates fatty acids for oxylipin biosynthesis [4,77]. Wound inducibility of patatin (or its mobilization by proteases) positions it as both a structural storage protein and a potential contributor to early defense signaling [77]. However, mounting defense responses requires diversion of amino acids and carbon skeletons away from storage into defense proteins, secondary metabolites (e.g., phenylpropanoids), and repair processes, introducing trade-offs: higher defense investment can reduce starch deposition and lower tuber yield/quality [78,79]. Thus, during pathogen attack and wounding, patatin plays a double role as a direct/indirect antimicrobial source and as a remobilizable N reservoir with consequences for both resistance and storage reserve retention.

### 6.4. Synthesis—From Stress to Patatin to Reserves to Performance: Open Questions

**Figure 4:**
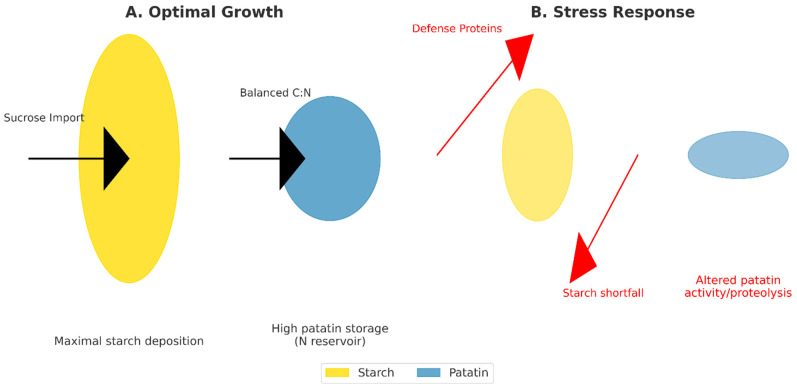
Two-panel model: (**A**) optimal growth maximal patatin + starch accumulation; (**B**) stress reallocation to defense, altered patatin abundance/activity, starch shortfalls.

As demonstrated in Figure 4 and Table 4, integrating the lines above suggests two central, testable ideas. First: stress-driven patatin degradation supplies amino acids for immediate repair and defense, at the cost of lowered long-term N reserves and potentially reduced starch deposition a scenario supported by proteomic and transcriptomic snapshots showing rapid proteome reprogramming and increased protease activity under abiotic stress and during sprouting [52,75,77]. Second: patatin-mediated lipid remodeling (PLA activity) produces oxylipins that feedback on carbon partitioning by altering plastidial signaling, redox state, or kinase cascades [4,71]. Both routes (N remobilization and lipid signaling) can operate concurrently, and their relative importance likely depends on stress type, intensity, developmental timing, and genotype. Critically, these hypotheses require experiments that combine targeted manipulation of patatin/pPLA activity (e.g., isoform-resolved CRISPR or inducible overexpression), high-resolution lipidomics (oxylipins), protease/proteome dynamics, and dual fluxomics (^13^C/^15^N) to quantify the trade-offs between defense/repair and storage [4,52,77]. Addressing these gaps will clarify whether patatin is primarily a passive store that is sacrificed under stress, an active signaling node that directs carbon allocation, or both and will inform breeding and management strategies to optimize tuber performance under increasing environmental stress.

## 7. Knowledge Gaps and Future Directions

### 7.1. Isoform-Specific Functions and Redundancy

Patatin is encoded by a multi-gene family with many closely related isoforms; this genetic redundancy complicates attribution of distinct physiological roles to individual isoforms (e.g., storage versus PLA-like signaling). Resolving isoform function will require allele- and isoform-aware strategies rather than whole-family knockouts. Modern polyploid-aware genetics (e.g., polyploid QTL-seq and ploidy-aware GWAS) and allele-specific transcriptomics can identify naturally occurring functional variation and dosage effects; these approaches should be combined with targeted, isoform-specific edits (CRISPR/Cas with allele-specific guides, base editing, or prime editing) and with precision phenotyping (starch composition, PLA activity, lipidomes) to dissect redundancy and neofunctionalization [84,85]. Functional assays must report not only total patatin abundance but isoform-resolved expression, post-translational modifications (e.g., phosphorylation) and subcellular partitioning, because small biochemical differences between isoforms may be the basis for divergent roles in storage versus signaling.

### 7.2. Need for Proximity Proteomics and Lipidomics

A persistent gap is the lack of spatially resolved, causal evidence that patatin (or patatin-derived lipids) directly affects amyloplast function or starch enzymes. Proximity labeling (TurboID/miniTurbo, APEX) coupled with high-accuracy MS now allows organelle contact-site proteomes and transient inter-organelle interactomes to be mapped in planta [86,87]. Applying TurboID tethered to PSV membranes (or to specific patatin isoforms) and performing parallel proximity lipidomics would reveal physical neighbors and lipid changes at PSV–amyloplast interfaces. Such datasets combined with inducible, reversible manipulations of patatin PLA activity and targeted lipid perturbations would provide the direct, causal evidence (loss/gain of proximity partners, lipid mediators, and changes in AGPase/GBSSI activity) needed to move beyond correlation [86,87].

### 7.3. Sugar/Hormone–Patatin Regulatory Circuitry Under Fluctuating Environments

The T6P–SnRK1 module is now known in molecular detail to link sucrose status to global metabolic reprogramming, and recent structural work explains how T6P controls SnRK1 activity [88]. However, patatin’s position in this circuitry (is it downstream of T6P, part of a feedback loop via N remobilization, or both?) remains unresolved in tubers. Time-resolved perturbation experiments (e.g., transient T6P elevation or SnRK1 modulation, combined with patatin isoform reporters) and quantitative hormone measurements (ABA, JA, cytokinins) across diurnal cycles and bulking stages will be essential. Integrative interactome studies that include SnRK1 targets and patatin proximity partners could reveal whether patatin abundance/activity is regulated predominantly at transcription, translation, localization, or proteolytic turnover in response to sugar and hormone fluxes [88,89].

### 7.4. Multi-Omics + Fluxomics Under Realistic Field Stresses; Diurnal and Developmental Resolution

Laboratory stress treatments are useful but can misrepresent field dynamics. The recent “one-shot” ^13^C/^15^N MFA approach and related nonstationary MFA frameworks permit simultaneous, quantitative tracing of carbon and nitrogen fluxes and are directly applicable to tuber systems [90]. Combining time-series isotope tracing (diurnal and developmental sampling) with proteomics, phosphoproteomics, lipidomics, and targeted metabolomics under realistic field stress regimes (drought, salinity, pathogen pressure) will quantify how patatin turnover contributes to C and N fluxes and whether its proteolysis supplies amino acids for defense or accelerates starch remobilization. Such integrative flux-resolved multi-omics experiments are now technically feasible and will provide the quantitative, mechanistic models the field needs [90,91].

### 7.5. GWAS/Pan-Genome Mapping of Patatin Loci vs. Tuber Quality and Stress Traits

Large-scale association studies and pan-genome resources for potato allow systematic mapping of patatin loci to agronomic variation (e.g., dry matter, specific gravity, amylose content, stress resilience). Polyploid-aware methods (polyploid QTL-seq, tetraploid GWAS) can detect allele dosage effects and cis-regulatory variants that control patatin expression or stress inducibility [84,92]. Integrating GWAS signals with expression QTL (eQTL) and protein-QTL (pQTL) layers ideally in multi-environment trials will prioritize causal variants and enable marker development for breeding [84,92]. Establishing standardized metadata and mapping conventions (reference genome versioning, allele nomenclature) will be critical for cross-study synthesis.

### 7.6. Standardized Stress Protocols and Cross-Study Comparability

A final, practical gap is methodological: inconsistent stress definitions, timing, severity, and sampling schemes limit comparability across studies. Proteome reprogramming work in potato highlights that abrupt vs. stepwise water-deficit regimes produce distinct molecular signatures [91], illustrating why standardized protocols (stress intensity, duration, developmental stage at treatment, environmental co-factors) are essential. The field should adopt community standards (stress descriptors, sampling timing, minimum multi-omics layers, and reporting of soil/irrigation/climatic context) so that GWAS, multi-omics, and functional studies are interoperable and meta-analyses are meaningful. Standardization will accelerate translation: breeding programs need robust, comparable trait–gene associations that hold across environments and years [89,91].

## 8. Conclusions

Patatin emerges as a dual-function hub in potato tuber biology, operating both as a major storage protein and as a regulatory node with lipid acyl hydrolase-like activity. Evidence from transcriptomics, proteomics, flux analyses, and targeted genetic perturbations highlights that patatin is deeply integrated into tuber carbon–nitrogen allocation. It contributes to sink strength not only by serving as a nitrogen reserve complementing starch, but also by influencing vacuolar osmotic capacity and signaling interactions that condition sucrose import and starch biosynthesis. Under abiotic stresses such as drought and salinity, as well as biotic challenges from pathogens, patatin abundance and activity shift in ways that affect both storage function and defense signaling. These changes often redirect carbon and nitrogen resources from starch deposition to protective mechanisms, explaining starch shortfalls and altered tuber quality traits under stress. Such findings reinforce patatin’s role as a stress-sensitive integrator at the crossroads of energy storage and adaptive responses. From an applied perspective, patatin is increasingly recognized as a biomarker and leverage point for breeding and biotechnology. Allelic variation, promoter architecture, and isoform-specific roles provide promising entry points for marker-assisted selection, gene editing, and promoter engineering aimed at stabilizing patatin and starch accumulation in climate-vulnerable environments. In parallel, agronomic strategies optimizing nitrogen and water supply could complement genetic improvements, reinforcing patatin’s stability and contribution to sink strength.

Therefore, patatin can be viewed as a storage-and-signal hub that shapes both tuber carbon allocation and resilience to environmental stresses. Future efforts integrating multi-omics, fluxomics, and field-level trials will be critical for translating this knowledge into practical innovations. By explicitly targeting patatin in breeding pipelines and engineering strategies, potato improvement can move toward cultivars that combine high starch yield with robust stress tolerance, advancing the agenda of climate-ready agriculture.

## Figures and Tables

**Table 2:** Studies connecting patatin levels to starch content/quality traits (dry matter, specific gravity, amylose/amylopectin ratios).

Approach	Patatin-Related Observation	Starch/Quality Trait Measured	Key Findings	Study
Systems biology & metabolic flux analysis	Patatin expression correlated with sink strength during tuber bulking	Dry matter and starch accumulation	Strong positive correlation between patatin abundance and tuber dry matter/starch levels	[55]
qPCR & physiological assays under drought stress	Stress suppressed patatin expression while starch biosynthesis was downregulated	Dry matter and starch quality	Reduction in patatin linked with decreased starch and tuber quality under drought	[56]
Transgenic manipulation of sucrose signaling	Altered SnRK1/T6P balance influenced patatin expression	Starch content and quality traits	Patatin expression tracked with sugar signaling changes that modulated starch accumulation	[57]
CRISPR-Cas9 mutagenesis of patatin isoforms	Knockout of class I patatin reduced starch accumulation efficiency	Starch yield, amylose/amylopectin ratio	Loss of patatin disrupted starch partitioning, lowering starch yield and altering composition	[58]

**Table 3:** Starch enzymes, regulatory nodes, and hypothesized connections to patatin (direct/indirect).

Enzyme/Node	Primary Function in Starch Biosynthesis	Known Regulatory Inputs/Control Points	Hypothesized Connection to Patatin (Direct/Indirect)	Representative Recent References
Sucrose synthase (SuSy)	Produces UDP-glucose, contributing to ADP-glucose pool for starch synthesis.	Regulated by sucrose flux, oxygen status, and sink strength.	Indirect: Patatin-mediated vacuolar storage may enhance sink demand, indirectly promoting SuSy activity.	[2,50]
Cell wall/vacuolar invertases	Cleave sucrose to glucose + fructose, establishing osmotic gradients.	Controlled by sucrose, invertase inhibitors, and developmental stage.	Indirect: Patatin affects vacuolar osmotic balance, influencing hexose signaling during bulking.	[2,50,59]
ADP-glucose pyrophosphorylase (AGPase)	Gateway enzyme producing ADP-Glc for starch synthesis.	Allosterically regulated by 3-PGA/Pi; redox and phosphorylation-sensitive.	Indirect: Patatin influences sugar signaling (T6P/SnRK1) and N status, modulating AGPase activation.	[65,71,72]
Granule-bound starch synthase I (GBSSI)	Catalyzes amylose biosynthesis in starch granules.	Expression and plastid targeting regulated by substrate availability.	Indirect: Patatin-driven sink demand alters ADP-Glc pools; lipid/oxylipin signals from PLA activity may influence plastidial GBSSI.	[70,73]
Soluble starch synthases (SS), Starch branching enzymes (SBE), Isoamylases (ISA)	Construct amylopectin and determine granule structure.	Isoform-specific expression and protein–protein interactions; regulated by phosphorylation.	Indirect: Patatin impacts ATP/redox/amino acid availability, influencing SS/SBE/ISA activity.	[70,73]
T6P/SnRK1 axis	Sugar-sensing module controlling anabolic vs. catabolic fluxes; influences AGPase.	T6P levels reflect sucrose status; SnRK1 regulates metabolic gene expression.	Indirect → direct: Patatin expression is sucrose-responsive and feeds back through N remobilization, affecting T6P/SnRK1 balance.	[2,50,59]

**Table 4:** Summary of stress studies (species/cultivar, stress regime, patatin readouts, starch/DM outcomes, yield/quality effects).

Species/Cultivar	Stress Regime	Patatin Readouts	Starch/DM Outcomes	Yield/Quality Effects	Reference
*Solanum tuberosum* cv. Désirée	Drought (greenhouse; moderate vs. severe)	Transcript & protein levels under severe drought; PLA-like activity (lipid remodeling)	Reduced starch accumulation, soluble sugars (osmoprotection)	Lower tuber DM; yield penalties under severe stress	[80]
*S. tuberosum* cv. Kufri Jyoti	Salinity (NaCl 100 mM, hydroponics)	Patatin protein stability compromised; partial proteolysis observed	Decline in starch content, reduced specific gravity	Lower dry matter; increased ionic stress damage	[81]
*S. tuberosum* cv. Atlantic	ABA treatment mimicking drought signal	Patatin gene expression induced early, but protein turnover accelerated	Transient starch decline, sucrose retention in tuber parenchyma	Tuber bulking slowed; altered partitioning	[82]
*S. tuberosum* (wild accession: *S. chacoense*)	Pathogen (*Phytophthora infestans*) infection	Patatin isoforms upregulated in leaves and tuber periphery; lipid mediators released	Starch deposition inhibited near lesions	Yield loss due to defense prioritization; patatin linked to hypersensitive response	[83]
*S. tuberosum* cv. Russet Burbank	Mechanical wounding (postharvest storage test)	Wound-inducible patatin proteins; proteolysis after 48 h	Starch hydrolysis stimulated at wound sites	Quality loss: sweetening and textural changes	[50]
*S. tuberosum* cv. Innovator	Combined drought + heat stress (field trial)	RNA-seq: patatin transcripts; stress chaperones	Significant starch shortfall; amylopectin/amylose ratio altered	DM reduction; fry color defects; yield	[58]

## Data Availability

Data are available on request.

## Supplementary Materials

The following supporting information can be downloaded at: https://www.mdpi.com/article/10.3390/biology15010029/s1, Figure S1: PRISMA flow diagram of study selection for the systematic review on patatin in potato tubers; Table S1: PRISMA 2020 checklist.
