# Supplementary material for: The Multifunctional Role of Patatin in Potato Tuber Sink Strength, Starch Biosynthesis, and Stress Adaptation: A Systematic Review
Source: Biology (Basel). 2025 Dec 24;15(1):29. doi: 10.3390/biology15010029 (PMC12784821; doi:10.3390/biology15010029)
Supplement: Supplementary file 1 [file biology-15-00029-s001.zip › biology-4047899-Supplementary-Figure 1. PRISMA flow diagram.pdf]

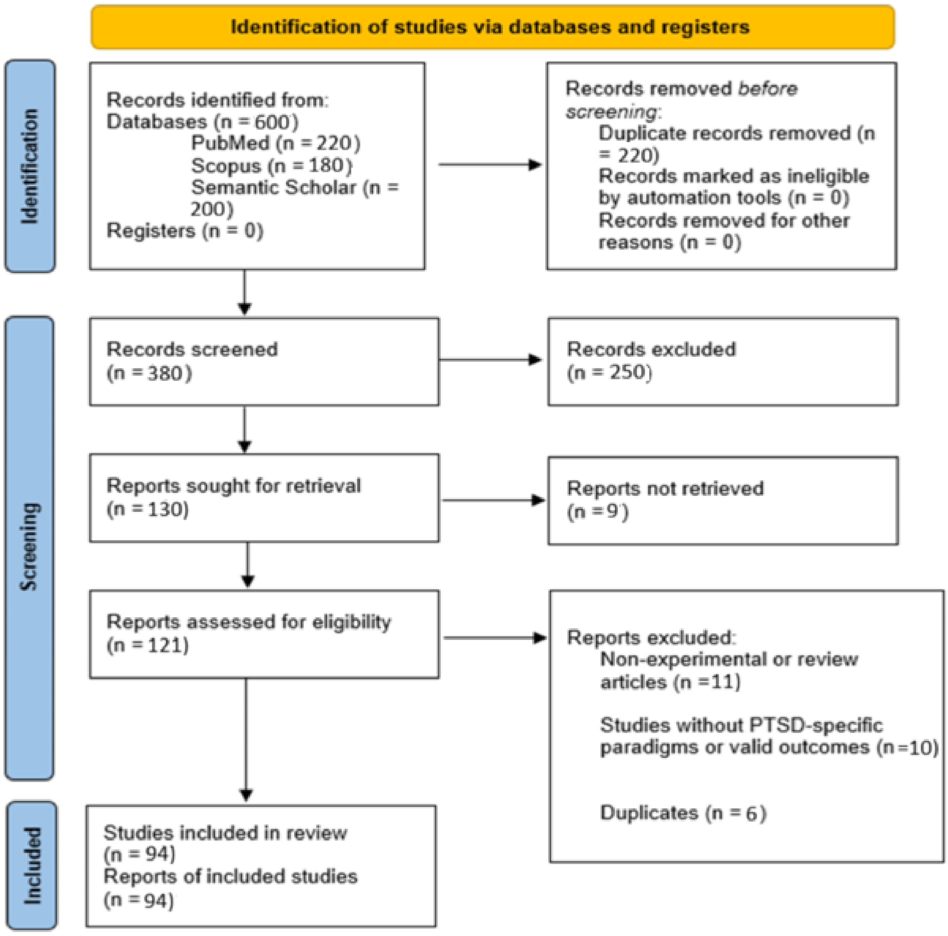

Supplementary Figure S1. PRISMA flow diagram of study selection for the systematic review on patatin in potato tubers. A total of 600 records were identified through database searches and reference lists. After removing 220 duplicates, 380 records were screened based on titles and abstracts. Of these, 121 full-text articles were assessed for eligibility, and 94 studies met the inclusion criteria. Studies were excluded for the following reasons: non-experimental/review articles (n = 11), lacking valid data and outcome (n = 10), and those with duplicate (6). The diagram illustrates the complete selection process from identification to inclusion.
